# The Expanded Exercise Addiction Inventory (EAI-3): Towards Reliable and International Screening of Exercise-Related Dysfunction

**DOI:** 10.1007/s11469-023-01066-2

**Published:** 2023-05-10

**Authors:** Umberto Granziol, Mark D. Griffiths, Liye Zou, Peiying Yang, Hannah K. Herschel, Annika Junker, Takayuki Akimoto, Oliver Stoll, Merve Alpay, Zeynep Aydın, Thomas Zandonai, Laura Di Lodovico, Mia Beck Lichtenstein, Mike Trott, Robert M. Portman, Melanie Schipfer, Brian Cook, Silvia Cerea, Aleksei Y. Egorov, Abril Cantù-Berrueto, Ricardo de la Vega Marcos, Paula Texeira Fernandes, Emilio Landolfi, Zsolt Demetrovics, Eliza E. Tóth, Marco Solmi, Attila Szabo

**Affiliations:** 1 Department of General Psychology, University of Padova, Via Venezia 8, 35131 Padua, Italy; 2 School of Social Sciences, Nottingham Trent University, Nottingham, UK; 3 Body-Brain-Mind Laboratory, School of Psychology, Shenzhen University, Shenzhen, China; 4 Faculty of Sport Sciences, Waseda University, Tokorozawa, Japan; 5 Martin-Luther University Halle-Wittenbergn, Halle (Saale), Germany; 6 Physical Education and Sports, Institute of Health Sciences, Sivas Cumhuriyet University, Sivas, Turkey; 7 International School of Advanced Studies, University of Camerino, Camerino, Italy; 8 Department of Pharmacology, Paediatrics and Organic Chemistry, Miguel Hernández University of Elche, Alicante, Spain; 9 Department of Social and Developmental Psychology, Sapienza University of Rome, Rome, Italy; 10 Institute of Psychiatry and Neuroscience of Paris (IPNP), Université Paris Cité, INSERM U1266, Paris, France; 11 Department of Clinical Research, University of Southern Denmark, Odense, Denmark; 12 Research Unit for Digital Psychiatry, Mental Health Services in the Region of Southern Denmark, Odense, Denmark; 13 Centre for Public Health, Queen’s University Belfast, Belfast, UK; 14 Centre for Applied Psychological Science and School of Social Sciences, Humanities & Law, Teesside University, Middlesbrough, UK; 15 St Augustine, USA; 16 Department of Biomedical Science, University of Padova, Padua, Italy; 17 Sechenov Institute of Evolutionary Physiology and Biochemistry of Russian Academy of Sciences, St. Petersburg, Russia; 18 University Autonomous of Nuevo León (UANL), San Nicolas de los Garza, Mexico; 19 Department of Physical Education, Sport & Human Movement, Autonomous University of Madrid, Madrid, Spain; 20 Department of Sport Science and GEPEN - Physical Education Faculty, State University of Campinas/UNICAMP, Campinas, Brazil; 21 School of Kinesiology, University of the Fraser Valley, British Columbia, Canada; 22 Institute of Psychology, ELTE Eötvös Loránd University, Budapest, Hungary; 23 Centre of Excellence in Responsible Gaming, University of Gibraltar, Gibraltar, Gibraltar; 24 Doctoral School of Psychology, ELTE Eötvös Loránd University, Budapest, Hungary; 25 Department of Psychiatry, University of Ottawa, Ottawa, ON Canada; 26 On track: The Champlain First Episode Psychosis Program, Department of Mental Health, The Ottawa Hospital, ON Ottawa, Canada; 27 Clinical Epidemiology Program, Ottawa Hospital Research Institute (OHRI), University of Ottawa, Ottawa, ON Canada; 28 Department of Child and Adolescent Psychiatry, Charité Universitätsmedizin, Berlin, Germany; 29 Institute of Health Promotion and Sport Sciences, ELTE Eötvös Loránd University, Budapest, Hungary

**Keywords:** Cross-cultural validation, Exercise addiction, Exercise Addiction Inventory, Exercise dependence, Measurement invariance

## Abstract

Exercise addiction (EA) refers to excessive exercise, lack of control, and health risks. The Exercise Addiction Inventory (EAI) is one of the most widely used tools in its assessment. However, the cross-cultural psychometric properties of the EAI could be improved because it misses three pathological patterns, including guilt, exercise despite injury, and experienced harm. Therefore, the present study tested the psychometric properties of the expanded EAI (EAI-3) in a large international sample. The EAI-3 was administered to 1931 physically active adult exercisers speaking five languages (Chinese, German, Italian, Japanese, and Turkish) and other measures for obsessive–compulsive behavior, eating disorders, and personality traits. The assessment structure and reliability of the EAI-3 were tested with factorial analyses and through measurement invariance across languages and sex. Finally, a cutoff point for dysfunction-proneness was calculated. The EAI-3 comprised two factors, reflecting the positive and pathological sides of exercise. The structure had excellent reliability and goodness-of-fit indices and configural and metric invariances of the scale were supported. However, three items caused violations in scalar invariance. The results of partial measurement invariance testing suggested an adequate fit for the data. Following sensitivity and specificity analysis, the EAI-3’s cutoff score was 34 out of a maximum score of 48. This preliminary study suggests that the EAI-3 is a promising tool for screening EA in an international sample, with a robust and reliable structure comparable across languages and sex. In addition, the proposed cutoff could pave the way toward a consensus on a threshold to screen for EA.

Exercise addiction (EA) is a behavioral addiction that has received increasing interest among researchers working in exercise science, medicine, and health psychology (Bircher et al., 2017; Demetrovics & Kurimay, 2008; González-Hernández et al., 2022), with over 1000 academic publications in this field of study (Szabo & Kovacsik, 2019). There is a consensus that EA occurs when there is a loss of control over exercise behavior that results in several negative consequences (Szabo, 2010; Szabo & Demetrovics, 2022). Individuals with EA commonly experience withdrawal symptoms and craving-like feelings if exercise is unfulfilled. Berczik et al. (2012) suggested that the craving for training in EA was comparable to the feelings associated with substance addiction. Accordingly, EA is considered a behavioral addiction (Rosenberg & Feder, 2014), similar to gaming, gambling, sex, shopping, and other nonsubstance-related addictions.

Apart from “gambling disorder” and “internet gaming disorder,” behavioral addictions do not appear in the fifth edition of the *Diagnostic and Statistical Manual of Mental Disorders* (DSM-5; American Psychiatric Association, 2013), the evidence-based medical reference source for psychiatric disorders. Moreover, while EA is at the higher end of a continuum from low to excessive exercise (Glasser, 1976; Sicilia et al., 2021), there appears to be no dose–effect relationship, with exercise volume appearing unrelated to EA (Szabo & Kovacsik, 2019).

Exercise addiction can be classified as primary or secondary. In primary EA, the reward is the fulfillment of the exercise, while in secondary EA, exercise is a means of achieving another goal (Weinstein & Szabo, 2023; Yang et al., 2022). For example, excessive uncontrolled exercise co-occurs with eating or body-image disorders as an emotion regulation strategy related to body shape and weight control (Kun et al., 2022). Therefore, EA is secondary to more severe clinical pathology. Consequently, EA can be primary or a symptom of other clinical conditions such as eating disorders (American Psychiatric Association, 2013; Reinboth et al., 2021). Therefore, the motivating source and exercise manifestation are highly heterogeneous in EA.

Empirical evidence suggests that EA can cause: (i) physical harm (e.g., cardiovascular complications, repeated fractures, and musculoskeletal injuries; Hausenblas et al., 2017; Lichtenstein et al., 2017; Wouthuyzen-Bakker & van Assen, 2015), (ii) psychological harm (Aidman and Woollard, 2003; Szabo et al., 2016), and (iii) social harm (Fernandez et al., 2020). Juwono and Szabo (2021) used these criteria to classify morbidity in 100 interned-based EA testimonials. These negative consequences could be enough to identify dysfunctionality (Hausenblas & Symons Downs, 2002a). However, as aforementioned, EA is currently not recognized as a distinct class of psychiatric disorder. The reason beyond its recognized status is insufficient evidence for consistency in symptoms and etiology (Szabo & Demetrovics, 2022).

While it may be preferable to use the term “risk of exercise addiction” (REA; Lichtenstein et al., 2021), this terminology does not imply dysfunctionality (Szabo & Demetrovics, 2022). This issue surfaces in EA assessment tools. For instance, currently used psychometric instruments are not diagnostic tools. While their high scores may reflect a “risk” or predisposition to morbidity or even actual dysfunctionality, such scores often may have no clinical relevance (Szabo et al., 2015). Moreover, the variability in what the high scores reflect on various instruments is a significant issue in the field (Sicilia et al., 2021, 2022). Therefore, there is a need for instruments with an established cutoff value that suggests dysfunctionality while screening for EA. However, such an instrument would not have a definite diagnostic value but, followed up with a clinical interview, could serve as the early diagnosis of problematic exercise behavior (Weinstein & Szabo, 2023). Unfortunately, most EA assessment tools lack such a cutoff value or are not translated and validated in multiple languages. For example, the Questionnaire to assess Exercise Dependence in Endurance Athletes (Hauck et al., 2020; Schipfer, 2015) reports cutoff values in evaluating the REA, yet it is not widely used as it is validated on endurance athletes and is only available in German.

Among several assessment tools that have been developed for screening EA, a recent review (Sicilia et al., 2022) highlights two instruments that conceptualize EA as a disorder and simultaneously define a set of specific components of such a condition: the Exercise Dependence Scale-Revised (EDS-R; Hausenblas & Symons Downs, 2002a, b) and the Exercise Addiction Inventory (Terry et al., 2004). The Exercise Addiction Inventory (EAI) assesses thoughts, attitudes, and behaviors associated with exercise. Both the original and revised (Szabo et al., 2019) versions of the EAI comprise six items, evaluated on a Likert scale (five points in the original version and six points in the revised instrument). The EAI investigates six components: (i) salience, the extent to which exercise is the most important activity in the individual’s life; (ii) withdrawal symptoms, which include negative emotions and feelings experienced when a decrease or a stop in exercise occurs; (iii) relapse, intended as a compensatory mechanism that brings an individual to exaggerate or recursively repeat the exercise routine after a break; (iv) tolerance, referring to the tendency to increase the amount of exercise to experience the same positive effects; (v) mood modification, the use of exercise to change the mood or any feeling consequent to exercise; and (vi) conflict, corresponding to the presence of concerns arising from the individual’s exercise. Therefore, the EAI collectively comprises all aspects of everyday living (relationships, education, and/or occupation).

The EAI has been tested on recreational and professional athletes and among different types of sports (Alcaraz-Ibáñez et al., 2022a, b; Basoglu, 2018; Nogueira et al., 2018; Simon Grima et al., 2018). Compared to other instruments, it is fast to administer and easy to interpret, and its results are comparable to the EDS-R results. Finally, its psychometric characteristics are good, and its structure is similar in different countries (Griffiths et al., 2015). It has been translated and adapted into Arabic (Syed et al., 2022), Chinese (Wang et al., 2022), Danish (Lichtenstein et al., 2014), English (Szabo & Griffiths, 2007; Warner & Griffiths, 2006), Hungarian (Szabo, 2021), Italian (Gori et al., 2021; Granziol et al., 2021), Mexican (Salazar et al., 2021), Persian (Akbari et al., 2022), and Spanish (Sicilia et al., 2013).

Despite its good psychometric properties, the EAI requires further refinement. For example, the EAI and EAI-R do not assess components like perseverance of harmful exercise despite problems such as pain, injuries, or medical advice to stop exercising. The lack of evaluation of such a component has been noted as a shortcoming in previous research on the EAI (Alcaraz-Ibáñez et al., 2022a, b; Granziol et al., 2021; Griffiths, 1997). The EAI also fails to capture the guilt associated with addictive behaviors, including EA (Alcaraz-Ibáñez et al., 2022a, b). The last component is the pervasiveness of negative consequences, namely the degree of physical, psychological, and social problems caused by a person’s rigid and compulsive exercise regime (Aidman and Woollard, 2003; Juwono & Szabo, 2021; Sicilia et al., 2021). Including these features in a revised version of the instrument can more adequately reflect addiction than the current instrument, which could yield confounding results with passion and exercise volume (Szabo & Kovacsik, 2019).

Generating an expanded version of the EAI requires testing its structure and, more importantly, its independence from general research-inherent variables, such as gender and language. For example, previous research has highlighted the need for more evidence in favor of measurement invariance across sex (Dumitru et al., 2018; Granziol et al., 2021). Likewise, the unique previous cross-cultural validation of the EAI (Griffiths et al., 2015) suggested that the single-factor structure of the EAI had configural and metric invariance (i.e., both the factor structure and the loadings of each item to the unique factor were the same across languages). However, scalar invariance (i.e., the starting average score of each item per language) was violated. Therefore, it needs to be determined whether an expanded version of EAI retains these properties. If it does, then the sources of such cultural differences should be identified. Therefore, the present study aimed to cross-culturally validate an expanded, presumably more sensitive version of the EAI, namely the EAI-3. The specific objectives were:

1. Evaluation of the overall reliability and factor structure of the EAI-3 on an international sample. It was hypothesized that the new scale would present similar or better psychometric properties than the previous six-item versions (Szabo et al., 2019; Terry et al., 2004).
2. Evaluation of the EAI’s validity (i.e., content, construct, and criterion validity).
3. Assessment of the cross-cultural validity of the EAI-3 by testing the measurement invariance of the revised scale on gender and, simultaneously, the individual’s primary language (Chinese, German, Italian, Japanese and Turkish). It was hypothesized that the EAI-3’s configural and metric invariance (at least) would be supported. In case of invariance violations, finding the source of noninvariance was planned.
4. Identification of a preliminary and internationally adoptable cutoff score of the EAI-3.

## Methods

### Procedure

Participants were recruited online through social media platforms (i.e., *Facebook*, *Instagram*, and *Twitter*). Groups with a high likelihood of interest in participation because of their sports or exercise subject affiliation were specifically targeted. Snowball sampling was also used to increase the number of participants. In particular, participants were asked to send the survey link to other individuals who exercised, after they completed the survey.

The study used the *Qualtrics* survey platform (Qualtrics, 2023). First, participants provided their informed consent. The dedicated form was the first page of the online survey and contained all the information regarding the study, its aims, and the information about the principal investigator. Participants were informed that they could leave the survey at any time and that their data would be treated as anonymous if they decided to participate. Information such as name, surname, and email was not asked for. Since data collection was planned to be anonymous, participants were asked to generate a random code associated with their responses to avoid duplicates. Participants received no payment or any other reward for participating in the study. The research was conducted according to the Declaration of Helsinki (World Medical Association, 2013).

The inclusion criteria were as follows: being aged 18 years or older, engaging in exercise three times per week or more, engaging in exercise for at least 150 min (on average) per week, and being regular engaged physical activity in the past 6 months (i.e., no period without physical activity). Such inclusion criteria were in line with the World Health Organization (2020) guidelines for physical activity. Exclusion criteria in the present study were as follows: being less than 18 years old, exercising less than three times per week, exercising less than 150 min per week, exercising for less than 6 months, missing to answer to any of the EAI’s items, and answering the entire survey in less than 7 min. This last criterion emerged from pilot trials suggesting that a reasonable time to complete the survey was, on average, 8 min (SD =  ± 1 min).

### Measures

#### Expanded Exercise Addiction Inventory

Three items were added to the EAI/EAI-R (Szabo et al., 2019; Terry et al., 2004). These items were added to assess the “feelings of guilt” derived from the personal exercise habits: the tendency to “train even when injured” and the experience of “negative consequences”. These items were based on empirical evidence of their association with EA (Alcaraz-Ibáñez et al., 2022a; Granziol et al., 2021; Griffiths, 1997; Juwono & Szabo, 2021). Only the word “conflicts” in item 2 was substituted with “concerns” to make such an item comparable for different cultures. All the new items were translated and back-translated following internationally recognized protocols (Beaton et al., 2000). The same applied to the original items if there was not available a validated version of the EAI-3 for a specific language.

More specifically, each item was translated in the target language by a native speaker with proficient knowledge of English. After that, each item was back translated from the target language to English (by a different person) with the same language proficiency. If the back-translated item was comparable (or almost equal) from a grammatical and semantic point of view, it was included in the final translated version Otherwise, the procedure was repeated with the involvement of a third researcher. The EAI-3 items can be found in the Appendix Table 5. As for EAI-R (Szabo et al., 2019), each item of EAI-3 is rated on a six-point Likert Scale, from 1 (*Strongly disagree*) to 6 (*Strongly agree*). The score is obtained by summing the items’ scores. Therefore, the score ranges from 6 to 54. A higher score reflects higher severity of the potential symptomatology.

#### Exercise Dependence Scale-Revised (Hausenblas & Symons Downs, 2002a, b)

The EDS-R is a 21-item scale, where each item (e.g., “I am unable to reduce how long I exercise”) is rated on a six-point Likert response scale from 1 (*Never*) to 6 (*Always*) with total scores ranging from 21 to 126 (*α* = 0.95; *ω* = 0.96). The EDS-R has seven sub-categories, each of them comprising three items: withdrawal (*α* = 0.84; *ω* = 0.85), continuance (i.e., the tendency to continue exercise despite having physical or psychological problems due to exercise; *α* = 0.84; *ω* = 0.85), tolerance (*α* = 0.89; *ω* = 0.89), control loss (i.e., the desire to reduce or stop exercise, unsuccessfully; *α* = 0.88; *ω* = 0.89), a decrease of other activities (*α* = 0.72; *ω* = 0.74), time (i.e., the amount of time spent in activity related to exercise; *α* = 0.89; *ω* = 0.89), and effect intention (*α* = 0.91; *ω* = 0.92). The overall score for each category is calculated by adding the ratings for each item. A higher score often denotes a greater likelihood of developing an EA. For each subscale, an individual is considered at risk if the score is greater than 14, nondependent but symptomatic if it is between 7 and 14, and nondependent but asymptomatic if it is less than 7. The EDS-R manual (Hausenblas & Symons Downs, 2002b) suggests an exercise-dependent profile when at least three subscales have an “at-risk” score, a nondependent-asymptomatic profile when at least four subscales have a nondependent-asymptomatic score, and a nondependent-symptomatic profile in all other cases. This scale was used as a “gold standard” to test the criterion validity of the EAI-3, since many studies showed its stability in investigating EA (di Lodovico et al., 2019). It should also be noted that the EAI and the EDS are the two most widely used scales in the field of EA (Szabo & Demetrovics, 2022).

#### The *SCOFF* questionnaire (Sick, Control, One stone, Fat, Food; Morgan et al., 1999)

The SCOFF is a scale screening for eating disorder symptomology. It comprises five dichotomously rated items (e.g., “Do you worry that you have lost control over how much you eat?”) with total scores ranging from 0 to 5, which is obtained by summing the items’ scores (*α* = 0.65; *ω* = 0.78). The SCOFF is a short and easy-to-use scale. A score equal to or higher than 2 suggests a potential risk of an eating disorder. The SCOFF has shown excellent sensitivity and specificity against clinically diagnosed eating disorder patients (Hill et al., 2010). This scale was used because EA and eating disorders frequently co-occur or are primary or secondary to each other (Alcaraz-Ibáñez et al., 2020; Trott, et al., 2020, 2021).

#### Obsessive–Compulsive Inventory-Revised (Abramowitz & Deacon, 2006)

The Obsessive–Compulsive Inventory-Revised (OCI-R) assesses the tendency toward obsessions and compulsive thoughts and behaviors. It comprises 18 items with six factors (washing, *α* = 0.85; *ω* = 0.85; obsessing, *α* = 0.84; *ω* = 0.85; hoarding, *α* = 0.74; *ω* = 0.75; ordering, *α* = 0.79; *ω* = 0.80; checking, *α* = 0.80; *ω* = 0.82; mental neutralizing *α* = 0.82; *ω* = 0.83). Each item (e.g., “I check things more often than necessary”) is rated on a five-point response scale from 0 (*Not at all*) to 4 (*Extremely*) with total scores (obtained by item’s score sum) ranging from 0 to 70 (*α* = 0.93; *ω* = 0.95). Higher scores indicate higher severity in obsessive and compulsive manifestation. As suggested by several authors (Gulker et al., 2001; Naylor et al., 2011), there is a relationship between EA and obsessions and compulsions.

#### Ten-Item Personality Inventory (Gosling et al., 2003)

The Ten-Item Personality Inventory (TIPI) assesses the big five personality domains (i.e., extroversion, *α* = 0.67; agreeableness, *α* = 0.18; openness to experience, *α* = 0.45; conscientiousness, *α* = 0.71; emotional stability, *α* = 0.61), investigated by two items (e.g., “I see myself as extraverted, enthusiastic”) rated on a seven-point Likert scale from 1 (*Disagree strongly*) to 7 (*Agree strongly*). The scoring of each domain (range: 2–14) is obtained by summing the first item’s score and the reverse score of the second item. This instrument was included because EA is associated with several Big Five personality traits (Bircher et al., 2017; Di Lodovico et al., 2018; Miller & Mesagno, 2014).

### Analytic Plan

The analytic plan consisted of three blocks of analyses: (a) factor analyses (both exploratory and confirmatory), (b) measurement invariance, and (c) reliability and validity of the EAI-3. For each block, a subsample was estimated. The sample size estimation and details about each block are described below. All the statistical analyses were performed using the software *R* (R Core Team, 2020). In general, for each sample size estimation, an *α* = 0.05 and a power (i.e., 1 − *β*) of 0.80 were used as fixed parameters to estimate each sample size a priori.

### Factor Analysis and Measurement Invariance

#### Exploratory Factor Analysis

Since the EAI-R-3 included three new items, an exploratory factor analysis (EFA) was performed. The number of factors was a priori estimated through parallel analysis by using the *nFactors* package (Raiche & Magis, 2022). The EFA model was calculated by using a maximum likelihood estimation procedure on a polychoric correlation matrix and oblimin rotation. For this analysis, a model with 27 degrees of freedom was assumed, and to obtain an RMSEA ≤ 0.05, 358 participants were needed (~ 72 per language). The following rationale was applied to decide on cross-loadings: whenever an item correlated ≥ 0.3 with both factors and the difference among correlations was ≤ 0.20, a cross-loading was considered for that item (Costello & Osborne, 2005).

#### Confirmatory Factory Analysis

The structure suggested by the EFA was tested using confirmatory factory analysis (CFA). Due to skewness of data, the CFA was run by adopting a weighted least squares estimator with robust standard errors and a mean- and variance-adjusted test statistic (using a scale-shifted approach, WLSMV; Li, 2016). In line with the suggestions of previous research (Mónok et al., 2012; Schermelleh-Engel et al., 2003; Weston & Gore, 2006), two goodness-of-fit statistics to examine the model’s fit were applied: the comparative fit index (CFI < 0.90 indicates a not acceptable fit; between 0.90 and 0.95 indicates adequate fit; CFI > 0.95 indicates a good fit; Hu & Bentler, 1999) and the root mean square error of approximation (RMSEA; estimates ≤ 0.05 indicate a good fit, estimates between 0.05 and 0.10 an adequate fit while estimates > 0.10 a not acceptable fit; Steiger, 1990). As Zhao (2015) suggested, CFI and RMSEA are the most reliable indices in the case of the WLSMS estimator. As with the EFA, 358 participants were needed (~ 72 per language). The same indices were estimated for both the previous versions of the EAI.

#### Measurement Invariance

To determine whether sex and spoken language might influence the participants’ responses, measurement invariance (MI) was tested, focusing on configural, metric, and scalar invariances (van de Schoot et al., 2012). As proposed by several authors (Cheung & Rensvold, 2002; Gilson et al., 2013), two criteria were focused upon to compare the two models’ fit and to discuss whether measurement invariance had been observable: (i) the difference (Δ) between fit indices of the models, where we considered a CFI > 0.01 and an RMSEA > 0.015 as indicative of invariance violations, and (ii) the overall fit of each model. As suggested by Rhemtulla et al. (2012), when the response scale contains more than five categories, the scalar invariance can focus on the differences among intercepts’ items. The *lavaan* package (Rosseel, 2012) was used to test all the models.

Whenever an item caused misspecification in both the model tested on several specific subsamples and the MI models’ comparison, the item was removed from the final version of the scale. Otherwise, partial measurement invariance was tested, relaxing only the parameters related to the critical item. In both cases, the choice was made after consulting the modification index. For all the MI models, 830 participants were necessary (~ 83 participants per group). Samples size was estimated using the *semPower* package (Moshagen, 2021).

### Reliability, Cutoff, and Validity

#### Reliability

Cronbach’s alpha (Cronbach, 1951) for internal consistency, McDonald’s Omega (McDonald, 1999), and composite reliability (Bacon et al., 1995) were applied to test reliability. For the former two indices, a value ≥ 0.70 suggested adequate reliability, while for the latter, a value ≥ 0.60 was sufficient (Bagozzi & Phillips, 1982; Diamantopoulos & Winklhofer, 2001). The same indices were used for both the previous versions of the EAI.

#### Cutoff Score Estimation

The receiver operating characteristic (ROC) curve was used to evaluate the specificity and sensitivity of EAI-3 cutoff. To choose the best EAI-3 cutoff, the categorization obtained by applying the EDS-R manual was used as external criterion. Participants who emerged with an “at-risk” profile were used to define the “at-risk” condition, compared to all the others that were considered as “not-at-risk.” Moreover, a preliminary cutoff was also estimated for participants who declared themselves professional athletes.

#### Other Analyses

With regards construct validity (and excluding both the structural and cross-cultural ones that were evaluated in the previous analyses), convergent validity was evaluated by correlating both EAI-3 total scale and subscales scores, with the OCI-R, SCOFF, and the more “pathological” scales of the TIPI. All the associations were tested using the Spearman correlation, given that it was expected that the scores would not be normally distributed. In line with previous studies (Alcaraz-Ibáñez et al., 2020; Gulker et al., 2001; Naylor et al., 2011; Trott, et al., 2020, 2021), an association between the new measure and the scales evaluating obsessive–compulsive and eating disorders was expected. The *DescTools* package (Signorell, 2023) was used to estimate Spearman’s rho coefficient and its 95% confidence intervals. Discriminant validity was tested by using the Heterotrait-Monotrait (HTMT) ratio of correlation (Henseler et al., 2015) on the factorial structure of the EAI-3. A value below 0.90 suggests appropriate discriminant validity (Gold et al., 2001). The *semTools* package (Jorgensen et al., 2022) was used to estimate the HTMT coefficient. For criterion validity (particularly concurrent), it was expected there would be a high association between EAI-3 total scale and subscales scores and EDS-R scores. Considering the high number of tests performed, the *p*-values were adjusted using a Bonferroni correction. Moreover, since the sample contained responses provided by individuals who reported some illnesses, potential differences between people reporting a diagnosis and people not reporting it were tested using simple regressions.

For these last three analyses, a sub-sample of 385 participants was used (~ 77 per language). This sample was sufficient to perform all the proposed analyses. The minimal sample size necessary to estimate a preliminary international cutoff point was 386 participants, considering the prevalence of the EA (roughly 3.4% in regular exercisers; Marques et al., 2019) and an area under the curve ≥ to.70. The *easyROC* shiny app was used (Goksuluk et al., 2016) for this estimation. Such a sample size is also adequate for correlational analyses. To estimate a correlation coefficient of at least 0.50, 336 participants were necessary. This sample size was estimated by using the *pwr* package (Champely, 2020).

## Results

### Participant Characteristics

In total, 1931 participants were recruited for the present study (43.50% female, 56.50% male), comprising five languages: Chinese, German, Italian, Japanese, and Turkish. The mean age was 32.24 years (SD = 16.25; range = 18–99). Participants declared that the aim of their exercise concerned their health (physical or mental; 57.12%), the mastery or improvement of their skills in the related sport (35.27%), or social reasons (7.61%). The primary exercise/sports practiced were aerobic (e.g., endurance, 23.15%), anaerobic (e.g., strength, 7.56%), or mixed (both aerobic and anaerobic, 69.29%). Slightly more than half of the participants perceived themselves as amateur/recreational exercisers (52.25%), while almost a quarter (23.46%) declared themselves to be professional athletes. The remaining quarter of the participants declared being neither amateur nor professional exercisers (24.29%). Approximately half of the participants (50.96%) performed individual exercise/sport, 27.76% performed team exercise/sport, and the remaining 21.28% performed both. Finally, 12.01% of the participants reported being diagnosed with a physical or mental disorder. Information on response rate per item and missing data for all the items of all the used tools are reported, for each block of analysis, in the Supplementary Materials. Only one individual did not complete the survey and their data were excluded from the analyses. Therefore, the final sample was 1930 participants.

### Factor Analysis

#### Exploratory Factor Analysis

Parallel analysis and the following EFA suggested two potential factors: The first comprised items investigating habits, relevance, and amount of exercise, which was named “health relevance” (HR). The second factor comprised consequences of excessive exercise, which was named “Addiction Tendency” (AT). Items’ correlation with each factor is displayed in Table 1 (left panel). Item 5 and Item 7 presented potential (but not relevant using the cutoffs previously introduced) cross-loadings.

**Table 1:** Results of exploratory factor analysis on the first sample subsample of data, with both eight and nine items

Item	HR	AT	HR	AT	Item wording
	9 items		8 items		
EAIR_1	**.48**		**.52**		1. Exercise is the most important thing in my life
EAIR_3	**.76**		**.76**		3. I use exercise as a way of changing my mood (e.g., to get a buzz, to escape, etc.)
EAIR_4	**.50**	.18	**.43**	.23	4. Over time I have increased the amount of exercise I do in a day
EAIR_6	**.52**		**.53**		6. If I cut down the amount of exercise I do and then start again, I always end up exercising as often as I did before
EAIR_2		**.53**		**.43**	2. Concerns have arisen between me and my family and/or my partner about the amount of exercise I do
EAIR_5	.39	**.48**	.16	**.65**	5. If I have to miss an exercise session, I feel moody and irritable
EAIR_7	.32	**.50**		**.79**	7. I feel guilty if I miss planned training or if my training does not go as well as planned
EAIR_8	.18	**.65**		**.72**	8. I am inclined to train when (or before completely recovered from) illness or injury
EAIR_9	-.20	**.71**			9. I have had physical, psychological and/or other issues due to my exercise regime

Bold values represent item-scale correlations that are both higher than 0.3 and have no issues of cross-loadings

*HR health relevance, AT addiction tendency*

### Confirmatory Factor Analysis and Measurement Invariance

#### Confirmatory Factor Analysis

**Fig. 1:**
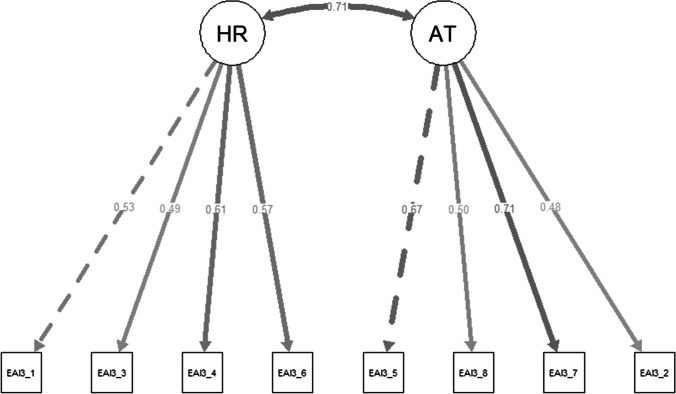
Results of confirmatory factor analysis. Dashed line describes the item presenting the first correlation with the subscale. HR, Health Relevance; AT, Addiction Tendency

The results of the CFA on the solution suggested by the previous EFA are displayed in Table 2. The model comprising two factors had issues in several cultural groups (Table 2). In fact, the model did not fit female participants speaking Chinese, Turkish, and Japanese, as for Turkish male participants. This result was reflected by low values of CFI and limit values of RMSEA. An examination of the modification indices suggested that items 1, 2, and 9 could cause misspecification and misfit issues. Therefore, separate models, removing one item at a time, were estimated. Only the removal of Item 9 led to an increase in all the estimates (see Table 3). An additional EFA model suggested adequate saturation of the other items (see Table 1). The new model yielded good to excellent fit indices in both overall sample and all specific groups. Therefore, only the model without item 9 (an 8-item model; Fig. 1) was analyzed subsequently. However, the previous versions of the EAI presented good fit indices (EAI: CFI = 1; RMSEA = 0; EAI-R: CFI = 0.99; RMSEA = 0.02).

**Table 2:** Results of the confirmatory factor analysis on the assessment model composed of nine items

Model	CFI	RMSEA	90% CI	Delta_CFI	Delta_RMSEA
Overall	.93	.060	.04–.08		
CH-F	.87	.07	.00–.122		
CH-M	1	.00	.00–.09		
IT-F	.98	.04	.00–.10		
IT-M	.98	.04	.00–.10		
TR-F	.89	.08	.03–.13		
TR-M	.85	.09	.04–.14		
JA-F	.85	.08	.00–.13		
JA-M	.96	.03	.00–.09		
GE-F	1	0	.00–.04		
GE-M	.95	.05	.00–.09		
Conf	.92	.06	.03–.09		
Met	.94	.05	.02–.07	.02	.01
Sca	.67	.11	.10–.12	.27	.06

*F* female, *M* male, *CH* Chinese, *IT* Italian, *TR* Turkish, *JA* Japanese, *GE* German, *Conf* configural model, *Met* metric model, *Sca* scalar model

**Table 3:** Results of the CFA and model invariance models

Model	CFI	RMSEA	90% CI	ΔCFI	ΔRMSEA
Overall	1	0	.00–.03		
CH-F	.98	.03	.00–.11		
CH-M	.99	.01	.00–.10		
IT-F	1	0	.00–.08		
IT-M	.95	.06	.00–.12		
TR-F	.96	.06	.00–.12		
TR-M	.98	.04	.00–.09		
JA-F	1	0	.00–.07		
JA-M	1	0	.00–.03		
GE-F	1	0	.00–.04		
GE-M	.99	.01	.00–.10		
Conf	1	0	.00–.05	.00	.00
Met	1	0	.00–.03	.00	.00
Sca	.73	.10	.09–.12	.26	.10
Par Sca (I1)	.91	.06	.04–.08	.09	.06
Par Sca (I1,2)	.96	.04	.00–.06	.04	.04
Par Sca (I1,2,7)	1	0	.00–.05	.00	.00

*Conf* configural model, *Met* metric model, *Sca* scalar model, *Par* partial, *I* item, *CH* Chinese, *IT* Italian, *TR* Turkish, *JA* Japanese, *GE* German, *F* female, *M* male

#### Measurement Invariance

The model presented both configural and metric invariance with excellent fit indices. Nonetheless, violations in scalar invariance were suggested (CFI = 0.74; RMSEA = 0.10; 90% CI = 0.09–0.12; ΔCFI = 0.26; ΔRMSEA = 0.10). Furthermore, the analysis of modification indices suggested that the intercept on item 1 (“Exercise is the most important thing in my life,” modification index = 62.33) could be different among groups, particularly among male participants speaking Japanese who presented higher scores on this item. Relaxing the parameters related to the item improved the model fit. Nonetheless, the violations remained (CFI = 0.91; RMSEA = 0.06; 90% CI = 0.04–0.08; ΔCFI = 0.09; ΔRMSEA = 0.06) and could be ascribed by a difference among intercepts of Item 2 (i.e., “Concerns have arisen between me and my family and/or my partner about the amount of exercise I do”; modification index = 19.72), in particular for male participants speaking Chinese, who presented higher average scores. Relaxing the parameters related to this item improved the model fit (CFI = 0.96; RMSEA = 0.04; 90% CI = 0–0.06; ΔCFI = 0.04; ΔRMSEA = 0.04), but still, some violations occurred. Another analysis on the modification indices suggested that the new Item 7 (“I feel guilty if I miss planned training or if my training does not go as well as planned,” modification index = 10.570) could have different intercepts, especially among female participants speaking German, who presented lower average score on this item. By relaxing the parameters related to this item, the model also presented scalar invariance (CFI = 1; RMSEA = 0; 90% CI = 0.00–0.05; ΔCFI = 0; ΔRMSEA = 0). A correlation matrix with the final set of the EAI-3 items is reported in the Supplementary Materials.

### Reliability, Cutoff, and Validity

#### Reliability

The overall score of the EAI-R-3 obtained adequate to good reliability indices (composite reliability = 0.81; Cronbach’s *α* = 0.81; McDonald’s *ω* = 0.84). Also, both the “Healthy Relevance” subscale (composite reliability = 0.66; Cronbach’s *α* = 0.70; McDonald’s *ω* = 0.74) and the Addiction Tendency subscale (composite reliability = 0.70; Cronbach’s *α* = 0.71; McDonald’s *ω* = 0.78) obtained acceptable reliability estimates. Both previous versions of the EAI also presented good reliability indices (EAI: composite reliability = 0.71; Cronbach’s *α* = 0.74; McDonald’s *ω* = 0.81; EAI-R: composite reliability = 0.71; Cronbach’s *α* = 0.74; McDonald’s *ω* = 0.84).

#### EAI-3 Cutoffs

**Fig. 2:**
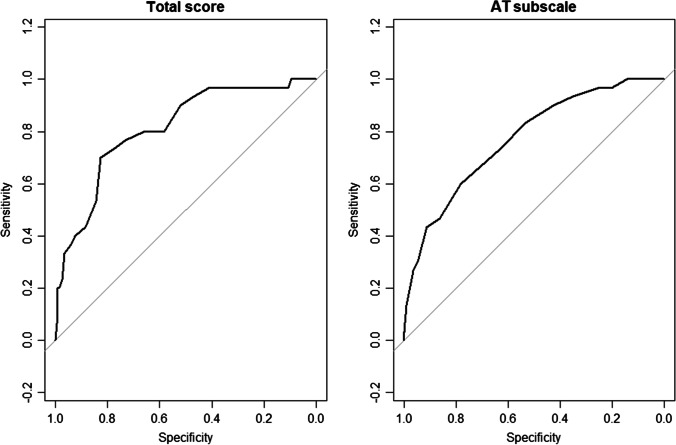
Results of analyses on receiver operating characteristic curve. The left panel shows the results on the total score. The right panel shows results on the Addiction Tendency subscale’s score. AT, Addiction Tendency

By applying the Exercise Dependency Scale-Revised categorization as a criterion (see previous section), 30 participants (12 F, 18 M) were classed as being at risk of EA. In comparison, 355 participants (137 F, 218 M) were not at risk. The analysis on ROC curves suggested that a cutoff of 33.50 out of 48 for the total score of the EAI-3 was sufficient to suggest a potential risk or susceptibility to EA. The specificity was 0.825, the sensitivity was 0.70, and the area under the curve (AUC) was equal to 0.812 (Fig. 2, left panel). Focusing only on the Addiction Tendency subscale, a cutoff of 15.5 out of 24 was sufficient to suggest a potential risk or susceptibility to EA. The specificity was 0.78, the sensitivity was 0.60, and the AUC was 0.77 (Fig. 2, right panel). Based on this new EAI-3 cutoff (total score), 83 participants out of 385 were considered at-risk of EA (21.6%). The kappa coefficient between the new cutoff and the one suggested by Hausenblas and Symons Downs was 0.29, indicating minimal agreement (Cohen, 1960).

Using the same analysis on the professional athletes’ subsample (*n* = 75) led to the same cutoff of 33.50. According to this cutoff, five out of 75 athletes were considered at-risk for EA (specificity = 0.71; sensitivity = 0.62; AUC = 0.66). Considering only the Addiction Tendency subscale, a cutoff of 19.50 emerged (specificity = 0.91; sensitivity = 0.37; AUC = 0.65).

#### Correlations with Other Measures

Table 4 contains the correlations between EAI-R-3 scores and the scores on the other psychometric scales.

**Table 4:** Results from correlational analyses

EAI-3	Measure	*p*	*p*-adj	95% CI
HR	EDS-R	.491	< .001	.41 to .56
AT	EDS-R	.597	< .001	.52 to .66
Total	EDS-R	.632	< .001	.57 to .69
HR	OCI-R (Tot)	.218	< .001	.12 to .31
AT	OCI-R (Tot)	.289	< .001	.19 to .38
Total	OCI-R (Tot)	.290	< .001	.19 to .38
HR	SCOFF (Tot)	.179	0.001	.08 to .27
AT	SCOFF (Tot)	.240	< .001	.15 to .33
Total	SCOFF (Tot)	.240	< .001	.14 to .33
HR	Extraversion	.039	.587	− .06 to .14
AT	Extraversion	− .035	.591	.13 to .07
Total	Extraversion	.006	.933	− .09 to .11
HR	Agreeableness	.033	.591	− .07 to .13
AT	Agreeableness	− .173	.002	− .27 to .07
Total	Agreeableness	− .088	.127	− .19 to .01
HR	Conscientiousness	.056	.381	− .04 to .16
AT	Conscientiousness	.093	.122	− .01 to .19
Total	Conscientiousness	.088	.127	− .01 to .19
HR	Emotional stability	− .090	.127	− .19 to .01
AT	Emotional stability	− .134	.018	− .23 to − .03
Total	Emotional stability	− .128	.024	− .23 to − .03
HR	Openness	.034	.591	− .07 to .13
AT	Openness	− .004	.933	− .10 to .10
Total	Openness	.027	.658	− .07 to .13

Bonferroni adjustment method was used

*HR* Health Relevance, *AT* Addiction Tendency

The EAI-3 total score and both subscales presented a positive correlation with the total numeric scores of both Exercise Dependency Scale-Revised (see Table 4), Obsessive–Compulsive Inventory-Revised, and SCOFF. In this last case, the Healthy Relevance subscale obtained lower correlation coefficients than the Addiction Tendency subscale, suggesting that the latter is more correlated to such a pathological construct. In addition, the Ten-Item-Personality-Inventory’s Agreeableness and Emotional Stability scores were negatively correlated with the Addiction Tendency scores. The EAI total score was negatively correlated with all the Emotional Stability traits. An appropriate discriminant validity was suggested by the HTMT analysis, with a value of 0.694.

Finally, to understand if reporting a disorder (physical or mental) or not could lead to different EAI-3 scores, linear models were tested, setting the EAI-R-3 scales’ scores as the dependent variable and the occurrence of a disorder as a predictor. Results suggested that individuals reporting a disorder did not differ in EAI-3 total scores (*β* = 1.45, *p* = 0.224, 95% CI: − 0.90 to 3.80), HR scores (*β* = 0.55, *p* = 0.386, 95% CI: − 0.70 to 1.79), or UR scores (*β* = 0.903, *p* = 0.204, − 0.49 to 2.30).

## Discussion

The present study examined the factorial structure, reliability, cross-cultural validity, and invariance of the nine-item EAI-3. Moreover, the present study aimed tried to identify an appropriate international cutoff for screening dysfunction-prone exercisers. Two factors were identified, describing the positive and pathological side of exercise. The EAI-3 showed excellent reliability and goodness-of-fit indices, with acceptable configural and metric invariance. The best cutoff estimate was 33.5. Since the scale does not allow decimal points, even a score equal to or higher than 34 can suggest the proneness to exercise addiction (EA).

The assessment of the risk of EA is essential because it helps to conceptualize a disorder that is becoming more widely researched, even without being officially formalized. The EAI is one of the most used instruments due to its brevity and easiness of use. Moreover, it contains items investigating an exhaustive set of manifestations of EA (Sicilia et al., 2021, 2022). The present study addressed some criticisms concerning the EAI, namely the need to add some components that define EA more precisely and to address measurement noninvariance in case of cross-cultural validation and the lack of an international cutoff.

To address the first issue, three novel items were added to the revised version of the EAI (Szabo et al., 2019). The first new item assessed the feeling of guilt that individuals can experience due to their exercise habits. Guilt is an emotion that can be found in sports at different levels. It can be found among coaches who perceive themselves as guilty when an injury occurs to the athletes they train (Martinelli et al., 2017). It can also be observed among athletes who make mistakes and, consequently, feel guilty for causing problems to their teammates (Jones, 2003).

Moreover, there are feelings of guilt caused by a perceived lack of training quantity or quality (Aidman and Woollard, 2003; Szabo et al., 1997). Finally, guilt could have a central factor in distinguishing secondary EA (Meyer et al., 2011). Another item assessed the tendency to exercise despite injuries or against medical advice. This item was related to the concept of continuance, which is frequently considered a component of EA (Hausenblas & Symons Downs, 2002a; Hausenblas et al., 2017). Finally, the last item assessed the perception of the negative consequences of excessive exercise (Berczik et al., 2012). Such consequences were identified in all 100 cases of EA analyzed by Juwono and Szabo (2021).

The exploratory factor analysis suggested that the assessment model provided by the new version of the EAI (EAI-3) could be better described by two factors: a first factor comprising aspects that are not necessarily related to a problematic exercise, such as the personal importance of the sport and the use of sport/exercise to change the mood or to break the routine. On the other hand, the second factor comprised aspects that more reflect a pathological use of the exercise. These include the presence of concerns and conflicts with family and friends, negative emotions (e.g., guilt) in case of missing training or if the quality of exercise is perceived as lower than a specific standard, the need to increase the quantity of exercise to perceive its effects (i.e., tolerance), and exercising despite the presence of injuries.

These additions and findings seem to meet the request of previous studies suggesting the need for psychometric instruments to be able to detect not only quantitative differences in the construct of EA, but also different patterns of responses or profiles (Blaydon et al., 2004; Magee et al., 2016; Sicilia et al., 2020). Consequently, it might be easier to discriminate between exercisers whose score is defined by a high commitment and passion for exercise and those who are addicted from a clinical perspective. Moreover, the presence of two factors that reflect two different ways of thought regarding exercise behavior is in line with theories of EA. Szabo and Demetrovics (2022) noted that some of the items of the EAI might be interpreted as negative while they are adaptive. For example, in a well-adjusted life of an elite athlete, high salience and other items can be considered ‘normal’. The two-factor structure of EAI-3 might be clinically helpful in discriminating potentially harmful exercise from actually harmful exercise, but this presumption needs further empirical investigation. Therefore, adding a nonproblematic exercise factor may offer the opportunity for the EAI-3 to be used as an unbiased brief clinical screening tool that may distinguish the wide breadth of benign exercise behavior patterns from those that also include potentially related aspects to addiction.

The confirmatory factor analysis of the EAI-3 also suggested that the assessment model that best fits the data should not consider the last of the three new items (i.e., item 9). This finding allows two interpretations. On the one hand, it could be that the item was formulated in a too generalist way, reducing the chance of detecting the specific intentions of the individual. For instance, the item investigated insight concerning physical, psychological, or social problems. On the other hand, it could be that the different languages and cultures of the participants could have created different ways of interpreting such an item, leading to the misfit problems that emerged from the analysis. Nonetheless, by removing the item, the structure of the EAI-3 emerged as adequate in all the five languages and sex, as suggested by the excellent goodness of fit indices, for both subscales and the overall score. This result was corroborated by the presence of configural and metric invariance, suggesting that the overall structure, the response patterns, and the items’ correlations to each factor were similar across language and sex.

However, the EAI-3 presented violations in scalar invariance. The source of such violations was observed on three items: the average score of item 1 (“Exercise is the most important thing in my life”) was higher for male Japanese participants, suggesting that they presented a higher starting level of the construct investigated by such an item (i.e., salience). This a priori difference should always be considered since it suggests that the baseline salience for these exercisers is generally higher. This is quite intuitive, considering the high commitment of Japanese individuals to sports activities (Hagiwara & Isogai, 2014). Beyond the specific result found in the present study, item 1 of the EAI has been frequently linked to problems with measurement invariance in previous studies (Alcaraz-Ibáñez et al., 2022a, b; Griffiths et al., 2015). Such coherence suggests that there could be some problems in defining the salience by using the current version of this item. Sicilia et al. (2022) suggest that a good approach to defining items assessing components of the EA should be written in a way that highlights the problematic nature of each EA symptom.

Similarly, the average score of item 2 (“Concerns have arisen between me and my family and/or my partner about the amount of exercise I do”) was higher for male Chinese participants, suggesting another cultural difference. Criticism of the item 2 among Chinese samples is not new. In fact, a recent study validating the EAI-R in a Chinese sample showed how the item presented low correlation to the factor structure and was removed (Wang et al., 2022). As suggested by the authors, this could be explained from a cultural point of view. In China, individuals typically consider exercise favorably, accordingly to a general belief that exercise is healthy. Consequently, family members may view it positively rather than disapproving of excessive exercise. Finally, it was observed that German female athletes reported lower scores on item 7 (“I feel guilty if I miss planned training or if my training does not go as well as planned”), contributing to the scalar invariance violations. This result suggests that female German athletes are more prone to stop exercising when injured and wait until they recover completely. Once the parameters related to such items were relaxed, the partial scalar measurement invariance was upheld.

Both the overall scale and the subscales showed acceptable reliability scores and correlations with other psychometric measures. The EAI-3 correlated moderately and positively with the EDS-R. Moreover, the positive relationship between EA and both obsessive–compulsive behavior and eating disorders was corroborated. Finally, the absence of a statistical and linear correlation between the Addiction Tendency subscale of the EAI-3 and Ten-Item-Personality-Inventory’s conscientiousness subscale and the negative correlation with agreeableness and emotional stability suggest that the pathological side of the exercise may be linked to more inner and potentially pathological aspect even in the personality of the exercises. A noteworthy result concerns the similarity between EAI-3’s fit and reliability indices and those of the previous versions of the EAI. This result suggests that EAI-3 can be compared to its antecedents, in terms of both structure appropriateness and reliable scores, with the advantage of investigating more aspects and, therefore, being more clinically accurate.

It is also worth mentioning the absence of a difference between the average scores of participants who declared to have received a medical or psychological diagnosis compared to participants without a diagnosis. This result suggests that the EAI-3 could be administered independently of whether an individual has a (physical or mental) health issue. Nonetheless, future studies should also test the measurement invariance of the EAI-3 with these two samples, to better understand if answering EAI-3 can be affected by having another disorder (physical or mental).

The present study also determined an initial international cutoff for the EAI-3 (i.e., 34). Differently from previous studies, such a cutoff could help discriminate between at-risk individuals and individuals not at risk of EA. In previous versions of the EAI (Szabo et al., 2019; Terry et al., 2004), a total score less than 13 suggested an asymptomatic profile; a total score between 13 and 23 suggested a potentially symptomatic profile, while a total score greater than 23 suggested at-risk profile (Griffiths et al., 2015). The present study suggests a simpler categorization (i.e., at-risk vs. non at risk, based on a total score of 34). The benefits of this could be easy screening for EA, especially if the aim is to find a cutoff that should be used worldwide. Potential drawbacks could concern the loss in precision if the aim is to distinguish between at-risk non asymptomatic and symptomatic. Since EAI-3 assesses the risk of EA, the solution proposed reduces such a criticism since the aim is to determine if the risk is present, not how much is present. Moreover, the cutoff estimated on the overall scale appeared to be stable, independently from the participants’ competition level (i.e., professional vs. amateur and noncompetitive exercisers). In fact, the same overall cutoff was obtained among professional athletes. However, the cutoff based on the Addiction Tendency subscale, on the contrary, emerged as higher for professional athletes. The different cutoff, in agreement with Szabo et al. (2015) and Szabo and Demetrovics (2022), suggests that athletes interpret the EAI items differently than others. Therefore, this issue merits further research attention. At present, this difference suggests that for professional athletes, a higher score on the Addiction Tendency subscale is needed to suggest the risk of EA.

### Limitations and Future Directions

The present study has some limitations. First, participants were recruited online and included self-report measures. Therefore, no other reported (and potentially less biased) information was collected and the data are subject to various methods biases (e.g., social desirability, memory recall). Moreover, the study was a cross-sectional survey conducted while the COVID-19 pandemic risks were still high. This prevented face-to-face recruitment and causality between variables could not be determined. Nonetheless, the use of validated and standardized self-questionnaires, that are brief and easy to use, allowed the collection of a large population size. Additionally, it could be argued that some exclusion criteria were too strict, such as eliminating responses generated in less than 7 min. Given the study used convenience sampling and snowball sampling, the response rate could not be determined because the research team had no idea how many individuals saw the online survey link or how many participants forwarded the link to other individuals. Also, the sampling method used means that the participants were not necessarily representative of all physically active individuals. Finally, it could be argued that violations in scalar invariance caused by three out of eight items represents an issue for a psychological assessment measure. This limitation also constitutes an advantage for future studies since it can lead to more accurate version of the EAI-3. In the present one, it was decided not to remove the items and test the partial invariance to understand where the differences among languages and sex occur. A plausible solution could be to remove, one at a time, all the items causing violations in scalar invariance and refit all the models.

In future research, it would be helpful also to estimate test–retest reliability on a smaller subsample given that this was not evaluated in the present study. Future studies could test both solutions among other languages to define the final version of the EAI-3. In fact, the present study can be considered as preliminary, and more cultures and languages need to be recruited and tested. In fact, the present study is part of a larger project including participants from Brazil, Canada, China, Croatia, Denmark, France, Germany, Hungary, Italy, Japan, Mexico, Norway, Portugal, Russia, Serbia, Spain, Turkey, Ukraine, the UK, and the USA. This will evaluate if the same results occur when these other languages are added in the measurement invariance models. A related future study could regard the ability of the EAI-3 to function in the same way considering the type of sport (e.g., aerobic, anaerobic, mixed; individual vs. team sports). Finally, it would be interesting to investigate which kind of information may be obtained from the use of EAI-3 among individuals that have high passion for sport or physical activity but that do not reach the criteria for exercise dependence or addiction.

## Conclusion

The results of the study suggest that the preliminary cross-cultural validation of the eight-item EAI-3 among male and female athletes coming from two European (i.e., Germany and Italy), one middle east Asiatic (i.e., Turkey), and two eastern Asiatic nations (i.e., China and Japan) is promising. The addition of new components suggested in scientific literature and an analysis of the source of possible differences among female and male athletes speaking different languages pave the way toward a more detailed and international component model of the exercise addiction.

### Electronic supplementary material

Below is the link to the electronic supplementary material.

Supplementary file1 (DOCX 35 KB)

## Data Availability

All the data used in this article can be available on request to the corresponding author.

## Appendix

**Table 5:** Initial items in the EAI-3

Items	Wording
Item 1	Exercise is the most important thing in my life
Item 2	*Concerns* have arisen between me and my family and/or my partner about the amount of exercise I do
Item 3	I use exercise as a way of changing my mood (e.g., to get a buzz, to escape, etc.)
Item 4	Over time I have increased the amount of exercise I do in a day
Item 5	If I have to miss an exercise session, I feel moody and irritable
Item 6	If I cut down the amount of exercise I do and then start again, I always end up exercising as often as I did before
*Item 7**	I feel guilty if I miss planned training or if my training does not go as well as planned
*Item 8**	I am inclined to train when (or before completely recovered from) illness or injury
*Item 9** ^±^	I have had physical, psychological and/or other issues due to my exercise regime

^*^Items 7–9 were newly added in the present study

^±^Item 9 is no longer part of the EAI-3
