# Supplementary material for: The Expanded Exercise Addiction Inventory (EAI-3): Towards Reliable and International Screening of Exercise-Related Dysfunction
Source: Int J Ment Health Addict. 2023 May 10:1–27. Online ahead of print. doi: 10.1007/s11469-023-01066-2 (PMC10171173; doi:10.1007/s11469-023-01066-2)
Supplement: Supplementary file 1 — Supplementary file1 (DOCX 35 KB) [file 11469_2023_1066_MOESM1_ESM.docx]

**Supplementary Materials**

**Response rates**

EAI-3 response rate for each point of the Likert scale. Data used for the exploratory factor analysis. Values refer to percentage.

|  | **Response scale points** | | | | | |  |
| --- | --- | --- | --- | --- | --- | --- | --- |
| **Item** | **1** | **2** | **3** | **4** | **5** | **6** | **Missing** |
| 1 | 4.75 | 10.61 | 15.08 | 28.49 | 25.42 | 15.64 | 0 |
| 2 | 29.61 | 31.01 | 8.38 | 16.48 | 10.06 | 4.47 | 0 |
| 3 | 4.19 | 9.5 | 9.78 | 24.02 | 32.4 | 20.11 | 0 |
| 4 | 5.59 | 20.95 | 17.6 | 27.65 | 21.23 | 6.98 | 0 |
| 5 | 10.06 | 24.3 | 18.72 | 29.33 | 12.01 | 5.59 | 0 |
| 6 | 3.63 | 14.8 | 15.64 | 30.45 | 26.82 | 8.66 | 0 |
| 7 | 7.26 | 18.44 | 12.85 | 30.17 | 20.67 | 10.61 | 0 |
| 8 | 14.25 | 23.46 | 13.41 | 25.14 | 18.44 | 5.31 | 0 |
| 9 | 34.64 | 28.21 | 12.85 | 13.97 | 6.15 | 4.19 | 0 |

EAI-3 response rate for each point of the Likert scale. Data used for the confirmatory factor analysis. Values refer to percentage.

|  | **Response scale points** | | | | | |  |
| --- | --- | --- | --- | --- | --- | --- | --- |
| **Item** | **1** | **2** | **3** | **4** | **5** | **6** | **Missing** |
| 1 | 3.07 | 11.73 | 10.89 | 29.61 | 31.01 | 13.69 | 0 |
| 2 | 35.47 | 29.33 | 7.26 | 15.64 | 8.38 | 3.91 | 0 |
| 3 | 3.63 | 9.5 | 5.87 | 31.84 | 30.73 | 18.44 | 0 |
| 4 | 5.03 | 21.51 | 17.32 | 26.54 | 18.99 | 10.61 | 0 |
| 5 | 13.13 | 28.21 | 14.53 | 30.45 | 10.61 | 3.07 | 0 |
| 6 | 6.7 | 14.8 | 18.16 | 28.77 | 24.86 | 6.7 | 0 |
| 7 | 9.22 | 13.69 | 11.73 | 34.92 | 24.02 | 6.42 | 0 |
| 8 | 14.8 | 20.95 | 12.29 | 28.77 | 18.44 | 4.75 | 0 |
| 9 | 36.87 | 29.89 | 12.29 | 11.17 | 6.98 | 2.79 | 0 |

EAI-3 response rate for each point of the Likert scale. Data used for the measurement invariance analysis. Values refer to percentage.

|  | **Response scale points** | | | | | |  |
| --- | --- | --- | --- | --- | --- | --- | --- |
| **Item** | **1** | **2** | **3** | **4** | **5** | **6** | **Missing** |
| 1 | 3.49 | 11.08 | 13.86 | 30.6 | 27.59 | 13.37 | 0 |
| 2 | 32.65 | 33.73 | 10.48 | 13.25 | 6.51 | 3.37 | 0 |
| 3 | 3.49 | 6.87 | 8.19 | 29.88 | 32.41 | 19.16 | 0 |
| 4 | 6.27 | 20.48 | 17.47 | 27.11 | 19.88 | 8.8 | 0 |
| 5 | 11.57 | 28.19 | 16.51 | 25.78 | 11.69 | 6.27 | 0 |
| 6 | 4.22 | 16.14 | 19.04 | 27.47 | 26.63 | 6.51 | 0 |
| 7 | 8.67 | 18.43 | 12.77 | 30.36 | 19.4 | 10.36 | 0 |
| 8 | 14.7 | 25.42 | 13.98 | 23.01 | 17.59 | 5.3 | 0 |
| 9 | 38.07 | 30.48 | 12.77 | 11.69 | 4.7 | 2.29 | 0 |

Response rate for each point of the Likert scale. Data used for all the remaining analyses. Values refer to percentage.

**EAI-3**

|  | **Response scale points** | | | | | |  |
| --- | --- | --- | --- | --- | --- | --- | --- |
| **Item** | **1** | **2** | **3** | **4** | **5** | **6** | **Missing** |
| 1 | 4.16 | 11.95 | 13.77 | 29.61 | 27.79 | 12.73 | 0 |
| 2 | 32.47 | 30.91 | 12.99 | 13.77 | 7.79 | 2.08 | 0 |
| 3 | 3.38 | 7.53 | 6.23 | 34.29 | 32.73 | 15.84 | 0 |
| 4 | 5.71 | 22.6 | 16.36 | 25.19 | 21.56 | 8.57 | 0 |
| 5 | 15.84 | 26.75 | 12.73 | 24.68 | 14.29 | 5.71 | 0 |
| 6 | 5.71 | 14.81 | 19.48 | 29.61 | 24.42 | 5.97 | 0 |
| 7 | 10.13 | 15.32 | 14.81 | 31.69 | 18.44 | 9.61 | 0 |
| 8 | 15.06 | 20.26 | 11.43 | 30.13 | 17.66 | 5.45 | 0 |

**EDS-R**

|  | **Response scale points** | | | | | |  |
| --- | --- | --- | --- | --- | --- | --- | --- |
| **Item** | **1** | **2** | **3** | **4** | **5** | **6** | **Missing** |
| 1 | 21.82 | 17.14 | 21.04 | 23.12 | 9.87 | 7.01 | 0 |
| 2 | 29.87 | 22.86 | 17.14 | 14.55 | 8.05 | 7.53 | 0 |
| 3 | 11.69 | 17.14 | 18.96 | 24.68 | 15.84 | 11.69 | 0 |
| 4 | 26.23 | 21.04 | 25.19 | 14.03 | 6.23 | 7.27 | 0 |
| 5 | 28.57 | 25.97 | 20.26 | 15.32 | 4.94 | 4.94 | 0 |
| 6 | 13.25 | 16.1 | 23.12 | 22.6 | 15.84 | 9.09 | 0 |
| 7 | 20.26 | 23.38 | 21.82 | 17.4 | 10.91 | 6.23 | 0 |
| 8 | 28.31 | 19.74 | 18.44 | 16.88 | 8.57 | 8.05 | 0 |
| 9 | 36.36 | 26.75 | 16.1 | 9.87 | 7.01 | 3.9 | 0 |
| 10 | 15.58 | 22.08 | 20 | 18.44 | 11.95 | 11.95 | 0 |
| 11 | 28.83 | 28.31 | 16.36 | 11.95 | 7.53 | 7.01 | 0 |
| 12 | 32.21 | 19.74 | 16.36 | 15.32 | 7.53 | 8.83 | 0 |
| 13 | 19.74 | 20 | 17.14 | 19.74 | 14.29 | 9.09 | 0 |
| 14 | 24.94 | 23.38 | 19.22 | 17.4 | 8.57 | 6.49 | 0 |
| 15 | 20.52 | 19.74 | 23.64 | 17.92 | 9.61 | 8.57 | 0 |
| 16 | 36.62 | 25.19 | 16.36 | 10.39 | 7.79 | 3.64 | 0 |
| 17 | 25.45 | 20.78 | 20.78 | 15.06 | 9.61 | 8.31 | 0 |
| 18 | 32.99 | 25.45 | 17.66 | 10.13 | 7.53 | 6.23 | 0 |
| 19 | 50.39 | 21.56 | 12.73 | 9.87 | 3.38 | 2.08 | 0 |
| 20 | 24.68 | 22.6 | 18.96 | 14.81 | 11.17 | 7.79 | 0 |
| 21 | 25.97 | 23.9 | 20.52 | 16.36 | 7.79 | 5.45 | 0 |

**SCOFF**

|  | **Response scale points** | |  |
| --- | --- | --- | --- |
| **Item** | **0** | **1** | **Missing** |
| 1 | 79.48 | 20.26 | 0.26 |
| 2 | 63.9 | 35.84 | 0.26 |
| 3 | 85.71 | 14.03 | 0.26 |
| 4 | 80 | 19.74 | 0.26 |
| 5 | 71.43 | 28.31 | 0.26 |

**OCI-R**

|  | **Response scale points** | | | | |  |
| --- | --- | --- | --- | --- | --- | --- |
| **Item** | **0** | **1** | **2** | **3** | **4** | **Missing** |
| 1 | 39.22 | 33.77 | 21.56 | 4.42 | 0.78 | 0.25 |
| 2 | 21.82 | 35.32 | 24.94 | 12.73 | 4.94 | 0.25 |
| 3 | 27.01 | 36.36 | 24.42 | 7.53 | 4.42 | 0.25 |
| 4 | 53.51 | 24.42 | 14.81 | 6.75 | 0.26 | 0.25 |
| 5 | 55.32 | 24.16 | 12.99 | 5.45 | 1.82 | 0.25 |
| 6 | 41.04 | 32.47 | 18.18 | 4.68 | 3.38 | 0.25 |
| 7 | 45.71 | 33.51 | 14.55 | 4.68 | 1.3 | 0.25 |
| 8 | 51.43 | 26.75 | 15.32 | 4.42 | 1.82 | 0.25 |
| 9 | 28.57 | 34.81 | 22.08 | 11.17 | 3.12 | 0.25 |
| 10 | 66.23 | 20.52 | 9.61 | 2.08 | 1.3 | 0.25 |
| 11 | 52.73 | 20 | 15.84 | 7.53 | 3.64 | 0.25 |
| 12 | 36.62 | 31.43 | 19.48 | 8.31 | 3.9 | 0.25 |
| 13 | 26.23 | 32.73 | 24.94 | 11.69 | 4.16 | 0.25 |
| 14 | 47.79 | 26.49 | 16.1 | 7.27 | 2.08 | 0.25 |
| 15 | 37.14 | 31.95 | 19.22 | 7.27 | 4.16 | 0.25 |
| 16 | 63.12 | 16.62 | 15.58 | 2.08 | 2.34 | 0.25 |
| 17 | 61.3 | 22.6 | 11.17 | 3.9 | 0.78 | 0.25 |
| 18 | 46.23 | 30.13 | 13.25 | 5.97 | 4.16 | 0.25 |

**TIPI**

|  | **Response scale points** | | | | | | |  |
| --- | --- | --- | --- | --- | --- | --- | --- | --- |
| **Item** | **1** | **2** | **3** | **4** | **5** | **6** | **7** | **Missing** |
| 1 | 2.86 | 7.01 | 11.43 | 15.32 | 21.3 | 22.6 | 19.22 | 0.26 |
| 2 | 22.08 | 21.82 | 11.69 | 15.06 | 22.86 | 3.9 | 2.34 | 0.26 |
| 3 | 1.82 | 2.6 | 8.05 | 12.99 | 21.04 | 27.27 | 25.97 | 0.26 |
| 4 | 14.55 | 16.88 | 16.88 | 11.69 | 25.97 | 9.09 | 4.68 | 0.26 |
| 5 | 1.56 | 2.86 | 10.65 | 8.83 | 25.19 | 28.31 | 22.34 | 0.26 |
| 6 | 15.32 | 14.29 | 16.36 | 16.88 | 15.58 | 15.58 | 5.71 | 0.26 |
| 7 | 0.78 | 1.04 | 3.9 | 10.65 | 23.64 | 32.47 | 27.27 | 0.26 |
| 8 | 22.6 | 18.18 | 10.91 | 16.1 | 20.52 | 8.83 | 2.6 | 0.26 |
| 9 | 2.6 | 4.94 | 12.99 | 15.84 | 27.27 | 25.19 | 10.91 | 0.26 |
| 10 | 12.47 | 14.03 | 19.22 | 18.96 | 20 | 10.39 | 4.68 | 0.26 |

**Correlation matrix of the EAI-3, final version.**

|  | EAI3-1 | EAI3-2 | EAI3-3 | EAI3-4 | EAI3-5 | EAI3-6 | EAI3-7 | EAI3-8 |
| --- | --- | --- | --- | --- | --- | --- | --- | --- |
| EAI3-1 |  |  |  |  |  |  |  |  |
| EAI3-2 | 0.19 |  |  |  |  |  |  |  |
| EAI3-3 | 0.31 | 0.21 |  |  |  |  |  |  |
| EAI3-4 | 0.31 | 0.22 | 0.29 |  |  |  |  |  |
| EAI3-5 | 0.23 | 0.3 | 0.23 | 0.34 |  |  |  |  |
| EAI3-6 | 0.3 | 0.21 | 0.27 | 0.29 | 0.22 |  |  |  |
| EAI3-7 | 0.25 | 0.28 | 0.27 | 0.26 | 0.52 | 0.31 |  |  |
| EAI3-8 | 0.21 | 0.27 | 0.05 | 0.23 | 0.3 | 0.25 | 0.38 |  |
